# Investigating changes of proteome in the bovine milk serum after retort processing using proteomics techniques

**DOI:** 10.1002/fsn3.2300

**Published:** 2021-12-30

**Authors:** Zikai Wei, Jiaxin Kang, Minhe Liao, Huanhuan Ju, Rong Fan, Jiaqi Shang, Xuenan Ning, Meng Li

**Affiliations:** ^1^ Key Laboratory of Dairy Science Ministry of Education Northeast Agricultural University Harbin China; ^2^ College of Food Science Northeast Agricultural University Harbin China

**Keywords:** bioinformatics, bovine milk, LFQ, proteomics, retort sterilized milk

## Abstract

The objective of this study was to investigate the changes of the proteins in bovine milk serum after retort processing by label‐free quantification proteomics techniques. A total of 96 and 106 proteins were quantified in control group (CG) and retort group (RG), respectively. Hierarchical clustering analysis of the identified milk serum proteins showed a decrease in the abundance of most proteins, such as serum albumin, lactoperoxidase, lactotransferrin, and complement C3, and an increase in the abundance of other proteins such as κ‐casein, lipocalin 2, and Perilipin. Student's *t*‐test showed 21 proteins significantly differential abundance between CG and RG (*p* < .05), of which intensity‐based absolute quantification (iBAQ) of five proteins decreased and iBAQ of 16 proteins increased. Bioinformatics analysis demonstrated that retort processing increased the digestibility of proteins, but this improvement was offset by a decrease in the digestibility of proteins caused by protein modification. Our results provide insight into the proteome of retort sterilized milk for the first time. Given the extremely high security of retort sterilized milk, the proteome of bovine milk serum changes after retort sterilization exposed in this study will contribute to the formula design of retort sterilized milk products.

## Graphical abstract

The objective of this study was to investigate the changes of the proteins in bovine milk serum after retort processing by label‐free quantification (LFQ) proteomics techniques. We discovered that while retort sterilization can increase the digestibility of denatured proteins in raw milk, this improvement was offset by a decrease in the digestibility of proteins caused by protein modification.

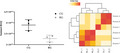

## 1 INTRODUCTION

Premature infant is defined as any born before a gestation of 37 completed weeks. Prematurity is the primary cause of neonatal mortality and is the second largest primary cause of death in children younger than 5 years (Blencowe et al., 2012). According to the preterm birth of fact sheets released by the Word Health Organization in February 2018, the rate of preterm birth ranges from 5% to 18% of babies born across 184 countries, and the preterm birth rates are increasing. In order to improve the life quality of premature infants, normative standard for premature infant feeding should be used. Generally speaking, human milk is considered to be the best option for feeding premature infants due to human milk is beneficial to improved neurodevelopmental outcomes; decreased risk of late‐onset sepsis, retinopathy of prematurity, and necrotizing enterocolitis (Bellando et al., 2020; Eidelman et al., 2012; Meredith‐Dennis et al., 2018; Ren et al., 2021; Vasquez‐Garibay et al., 2019). In North America, donor human milk (DHM) will be provided by Human Milk Banking Association of North America to premature infants when the mother’s milk is unavailable (Lima et al., 2018). However, DHM’s sterilization method is Holder pasteurization (sterilization at 62.5°C for 30 min). It is proved experimentally that *Bacillus cereus* remained in as many as 25% of processed milk samples after Holder pasteurization and it can lead to intestinal infections in infants, thereby causing a potent risk to infants, especially premature infants (Decousser et al., 2013). Therefore, a safer sterilization method is proposed for sterilization of raw milk, that is, retort sterilization (canned milk is heated to 121°C for 5 min). Compared with Holder pasteurization methods, retort sterilization completely eliminates heat‐stable toxins and spores and thereby offers a dramatically decreased risk profile (Meredith‐Dennis et al., 2018).

While retort sterilized milk can serve as a substitute for human milk to ensure the healthy growth of premature infant, since retort sterilized milk receives far more heat than ultra‐high‐temperature (UHT) milk and pasteurized milk during the sterilization process, the nutritional value of retort sterilized milk is lower than that of UHT milk and pasteurized milk, which is the reason for the low market share of retort sterilized milk (Anema, 2019; Takeda et al., 2015). Therefore, it is necessitated to take measures to learn about the changes of nutrients in bovine milk during retort processing. Proteins are tremendous nutritional value and are essential for life, but protein changes in milk after retort processing are still unknown. Under these circumstances, it is necessary to have a complete description of protein changes in retort sterilized milk. Proteomics is recognized as being a powerful tool for studying proteins in dairy products (Arena et al., 2017; Gagnaire et al., 2009; Lambers et al., 2015; Yang et al., 2019). For instance, in Greek sheep and goat milk serum, an average of more than 500 proteins were identified using cutting‐edge proteomics methodologies, which is the first time reporting the protein groups of milk from this valued biological material (Anagnostopoulos et al., 2016), and the proteins of goat milk during different heated processing were compared, revealing that heated processing enhanced the protein digestibility, which is beneficial to antiatherosclerosis therapy (Chen et al., 2019).

Given the safety of retort sterilized milk is extremely high, which is significant for premature infants, as well as the fact that the proteome of retort sterilized milk remains unclear. The objective of this work was to investigate the changes of proteome in bovine milk serum after retort processing. In present study, we simulated the sterilization process of retort sterilized milk in the laboratory and label‐free quantification (LFQ) technology was used for characterization of retort sterilized milk proteome. In addition, the concentration of β‐lactoglobulin in bovine milk serum was determined by enzyme‐linked immunosorbent assay (ELISA) and used to ensure the correctness of the proteomic results. The results of this study contribute to our understanding of retort sterilized milk and provide a theoretical basis for the design and optimization of retort sterilized milk formulations.

## 2 MATERIALS AND METHODS

### 2.1 Sample collection and preparation

Bovine milk was purchased from dairy farm in Harbin. The three layers medical gauze were used to filter the bovine milk to remove impurities (hair, particulate matter visible to the naked eye, etc.) from the milk. Then the milk samples were divided into two groups, a total of six samples. The samples of the first group were not heated and acted as the control group (CG), and the samples of the second group (180 ml each) were placed in glass tubes and heated in an autoclave (Zealway) at 118°C for 7 min and acted as retort group (RG). The procedure of the separation of milk serum proteins was as follows (Lu et al., 2018): firstly, samples were centrifuged at 1,500 *g* for 10 min at 10°C to remove the fat; secondly, skim milk was centrifuged at 10,000 *g* for 30 min at 30°C (Sichuan Shuke Instrument Co., Ltd.) to pellet the caseins. The protein concentrations of milk serum were determined by bicinchoninic acid (BCA) assay (Beyotime Biotechnology) and regulated so that an equal amount of protein was used for protein digestion.

### 2.2 SDS‐PAGE of milk serum proteins

SDS‐PAGE was used to characterize the influence of retort treatment on the major milk serum proteins in bovine milk. The concentration of milk serum proteins was diluted to 1 mg/ml according to the results of BCA measurement, and 10 μl of the diluted milk serum proteins was added to the SDS‐PAGE Sample Loading Buffer (Beyotime Biotechnology). Each milk serum sample was run on a 5% stacking gel at 80 V and a 12% separating gel at 120 V. Coomassie Brilliant Blue was used to stain the protein bands, which were then decolorized by placement in solution with 30% methanol and 10% acetic acid.

### 2.3 The determination of β‐lactoglobulin in milk serum proteins by ELISA

The determination of β‐lactoglobulin concentration in the samples was performed by means of the instructions included in the Bovine β‐lactoglobulin ELISA kit (ML Biotechnology). In brief, standard well and testing sample well were set. Standard (50 μl) was added to standard well, and milk serum sample (10 μl) and sample dilution (40 μl) were added to testing sample well. After treatment with incubate, configurate liquid, washing, add enzyme, color, and stop the reaction, the absorbance of three replicates was recorded by a Multi‐mode microplate reader (SpectraMax i3x，Molecular DEVICES) at 450 nm.

### 2.4 Protein digestion

Digestion method was described by previous articles, and the desalination method of sample with modifications (Ding et al., 2013). In brief, diluted milk serum (about 200 μg protein) was dissolved in 15 μl of 0.1 M dithiothreitol for 30 min at 56℃ and then alkylated by adding 15 μl of 0.5 iodoacetamide for 30 min at room temperature (25 ± 2°C) in the dark. After that, the sample was transferred to an ultrafiltration centrifuge tube (10 kDa, Millipore, cat.no. UFC501008) and centrifuged at 14,000 *g* for 20 min at 4°C. Then add 100 μl of 0.05 M NH_4_CO_3_, 14,000 *g* for 20 min at 4°C and repeat this step twice. The protein samples were digested by trypsin (Mass Spectrometry Grade, Thermo Scientific, cat.no. 90057) overnight at 37°C, and the mass ratio of enzyme/trypsin was 1:50, and the obtained peptides were collected as filtrate. The peptides of each sample desalted using C18 cartridge (Sep‐Pak, Waters, cat.no. WAT054960). The desalted samples were collected and concentrated to dryness in a vacuum centrifuge concentrator. Then, it was resolubilized using 0.1% formic acid and prepared for mass spectrometry analysis.

### 2.5 Liquid chromatography tandem mass spectrometry (LC/MS)

The setting of LC/MS was described as previously, and the gradient elution profile with modifications (Lu et al., 2018; Meredith‐Dennis et al., 2018). Digested protein samples were injected into a reversed‐phase trap column (Thermo Scientific Acclaim PepMap100, 100 μm × 2 cm, nanoViper, C18, 5 μm, 100 Å) followed by separating over a C18 reversed‐phase analytical column (Thermo Scientific Easy Column, 10 cm long, 75 μm inner diameter, 3 μm resin). Mobile phase A consisted of 0.1% formic acid in water, and Mobile phase B consisted of 0.1 formic in 80% acetonitrile; a set of linear gradients eluted in LC with a flow rate of 300 nl/min controlled by IntelliFlow technology: 0 ~ 5 min, 0%~8% B; 5 ~ 75 min, 8%~32% B; 75 ~ 77 min, 32%~90% B; 77 ~ 82 min, 90% B; 82 ~ 85 min; 90%~0% B; 85 ~ 90 min, 0% B. Mass spectrometric data were acquired using data‐dependent acquisition mode. The survey scan was from 350–1,800 m/z with resolution 70,000 at 200 m/z. The LTQ normal scan mode was used for the MS^2^ spectra.

### 2.6 Data analysis

MaxQuant v1.6.7.0 with Andromeda search engine was utilized to process the MS raw data and to search against the database of Bos taurus downloaded from UniProt (https://www.uniprot.org/, the databases were downloaded on 20‐09‐2019). Enzyme was defined as trypsin. Carbamidomethyl (C) was defined as fixed modification, and oxidation (M) and acetyl (N‐term) were defined as variable modification. Two missed cleavage sites were allowed, and mass match tolerance of 20 ppm and first and main search peptide were set to 20 ppm and 6 ppm, respectively. The false discovery rate for peptide and protein was set to 0.01 (Lu et al., 2018). To obtain absolute protein amounts, intensity‐based absolute quantification method (iBAQ) was used, which is the sum of intensities of all peptide peaks divided by the number of theoretically observable peptides (Schwanhausser et al., 2011). The iBAQ value can be represented as an indication for the absolute protein quantification (Zhang et al., 2016).

Perseus v1.6.7.0 was used for analyzing the proteinGroups.txt file obtained after MaxQuant processing. The protein iBAQ value was converted by log2(*x*), and the missing value treatment method was the normal distribution. The parameters were as follows: width: 0.3; downshift: 1.8. Two‐way Student’s *t*‐test was used for analyzing significant differences in the quantitative results, and the significantly different proteins were visualized using TBtools (Chen et al., 2018).

### 2.7 Bioinformatic analysis

DAVID v6.8 was used to analyze the gene ontology (GO) enrichment of significantly different proteins (Huang et al., 2009).

## 3 RESULTS AND DISCUSSION

### 3.1 Concentration and SDS‐PAGE of milk serum proteins

**FIGURE 1:**
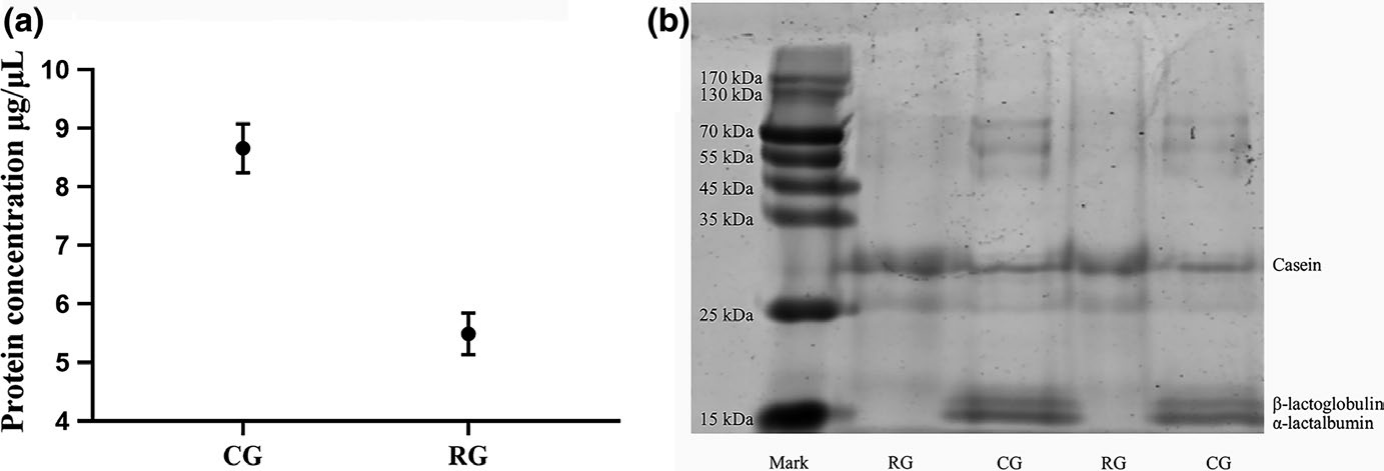
Protein concentration (a) and SDS‐PAGE (b) of milk serum proteins in CG and RG. CG represents unheated bovine milk serum proteins, RG represents bovine milk proteins heat‐treated by retort. The presentation of the values (a) is indicated by mean ± standard deviation (*SD*) of triplicate samples

The milk serum protein concentration and SDS‐PAGE of CG and RG are shown in Figure 1a and Figure 1b, respectively. It can be seen from Figure 1a that the concentration of milk serum proteins of CG was significantly higher than that of RG. From Figure 1b, it can be seen that the intensity of bands of α‐lactalbumin and β‐lactoglobulin was significantly weakened after retort processing. The heat treatment can denature bovine milk serum proteins, which then leads to a decrease in milk serum protein concentration and a weakening of the intensity of the major milk serum protein bands in bovine milk such as α‐lactalbumin and β‐lactoglobulin (Qian et al., 2017). Furthermore, when milk serum proteins were heat‐denatured, the sulfhydryl groups and disulfide bonds contained inside its molecules would be exposed, and protein aggregates could be formed through the exchange of sulfhydryl–disulfide bonds (Liu et al., 2018). Although these protein aggregates exist in the soluble state in bovine milk, a part of them was removed during high‐speed centrifugation, which leads to a decrease in milk serum protein concentration and a weakened intensity of the α‐lactalbumin and β‐lactoglobulin bands (Corredig & Dalgleish, 1996, 1999).

### 3.2 Identification and quantification of milk serum proteins

**FIGURE 2:**
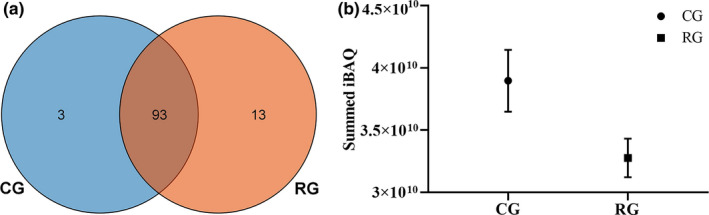
Venn diagram of quantified milk serum proteins (a) and summed iBAQ (b) in CG and RG. The Venn diagram shows the number and overlap of proteins identified in CG versus RG. The summed iBAQ represents the amount of protein abundance in the CG and RG. The presentation of the values (b) is indicated by mean ± standard deviation (*SD*) of triplicate samples

In present study, a total of 96 and 106 proteins were quantified in CG and RG, respectively. There were 93 co‐identical proteins in the CG and RG, and the characteristic proteins of CG and RG were 3 and 13, respectively, in Figure 2a. The number of identified proteins in bovine milk increased after retort processing. This result was similar to Chen’s result (Chen et al., 2019) which showed the change in the number of quantified proteins in heated goat milk. The reason for this situation is heat treatment increased the protein modifications at the molecular level (Gathercole et al., 2017). Protein modifications that occur in bovine milk during heat treatment usually have a negative impact on its nutritional properties, such as the ε‐amino group of the lysine (Lys) residue of the protein side chain can be modified to Nε‐carboxymethyllysine, a type of lysine that reduces the digestibility of the protein (Zhu et al., 2020), thereby decreasing the digestibility and nutritional value of bovine milk proteins. According to the previous report, heat treatment can improve the digestibility of proteins in raw milk through denaturation, but this improvement may be offset by a decrease in protein digestibility due to protein modifications (Wada & Lonnerdal, 2014).

In the present study, the iBAQ value of all proteins quantified in CG and RG was summed, and the results are shown in Figure 2b. The total iBAQ value of CG and RG should be theoretically equal since the amounts of milk serum proteins used for mass spectrometry analysis were equal, but the total iBAQ value of RG was lower than that of CG. This may be explained by the fact that the protein modifications reduced the digestibility of milk serum proteins in RG, resulting in a lower iBAQ value of RG than that of CG in the mass spectrometry analysis. This result was similar to previous research in that while heat treatment can increase the digestibility of denatured proteins in raw milk, this improvement was offset by a decrease in the digestibility of proteins caused by protein modification (Wada & Lonnerdal, 2014). The denaturation of bovine milk proteins caused by heat treatment was usually under consideration in the design of formulas. Nevertheless, the protein modifications that occur during heat treatment were often overlooked. In order to design products that more appropriately meet the needs of infants, measures such as reducing the heating temperature and time should be used to eliminate the adverse effects resulting from protein modifications.

### 3.3 Hierarchical clustering of milk serum proteins

**FIGURE 3:**
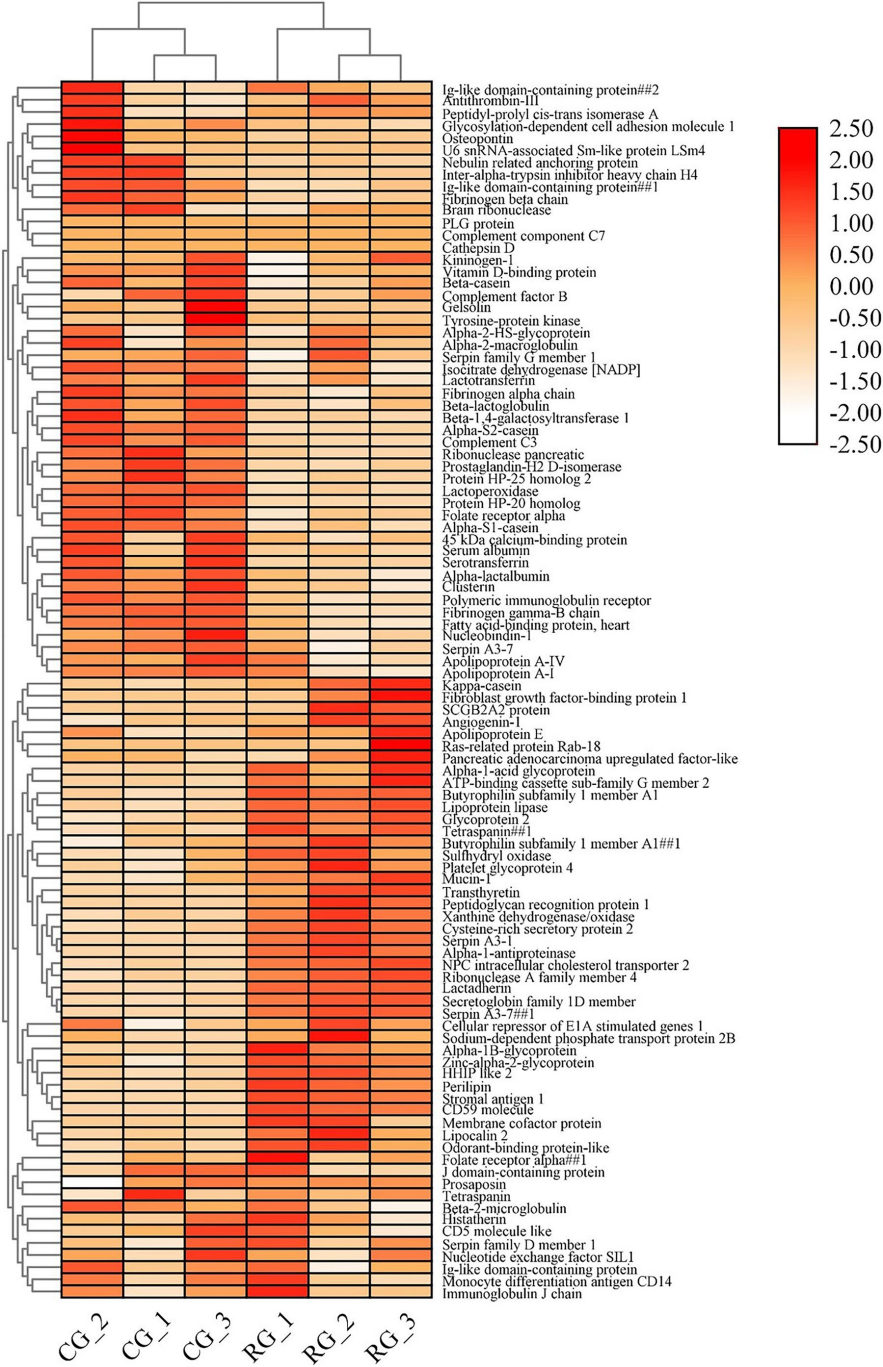
Hierarchical clustering of quantified milk serum proteins in CG and RG. Clustering according to protein abundance (iBAQ value by LC‐MS analysis) in CG and RG. Bar color represents a logarithmic scale from −2.50 to 2.50

The phenomenon of denaturation and protein modifications that occurs during heat treatment of milk serum proteins leads to changes in the composition of milk serum proteins. In order to visualize the changes that occur in the composition of bovine milk serum proteins during retort sterilization, milk serum proteins identified in CG and RG (proteins with the name uncharacterized protein and proteins with more missing protein iBAQ values were removed) were analyzed by hierarchical clustering using TBtools, and the results are shown in Figure 3. As can be seen in Figure 3, the abundance of most proteins was reduced after the retort sterilization, such as serum albumin, lactoperoxidase, lactotransferrin, and complement C3. This result was similar to that of Brick (Brick et al., 2017), who explored the effect of heat treatment on bovine milk serum proteins and found that heating significantly reduced biologically active proteins in bovine milk serum proteins, such as immunologically active lactotransferrin and complement protein C3, and antimicrobially active lactoperoxidase. Proteins like lipocalin 2 and perilipin increased in abundance during retort sterilization. The reason for this partial increase in protein abundance may be attributed to the fact that the amounts of proteins used for mass spectrometric analysis were equal for CG and RG in this study, and a decrease in the abundance of proteins such as α‐lactalbumin, β‐lactoglobulin, and lactotransferrin in RG would increase the abundance of other proteins. It can be hypothesized that proteins with increased abundance were more stable to heat compared to proteins with decreased abundance. It should be noted that this study was conducted on milk serum proteins, but casein was also identified in the mass spectrometry analysis, which is explained by the reason that a small fraction of casein remains free in milk serum proteins during the removal of casein by centrifugation. Among the identified caseins, α_S1_‐casein, α_S2_‐casein, and β‐casein decreased in abundance, while κ‐casein increased in protein abundance during retort sterilization. This may be a consequence of the easiness of κ‐casein to bind to other caseins to form micellar structures, which were partially disrupted during retort sterilization, thereby increasing the amount of free κ‐casein in milk serum proteins (Rehan et al., 2019).

### 3.4 Analysis of significant different proteins

Perseus software was used for data analysis of CG and RG obtained from MaxQuant software and used to find the significantly different proteins between CG and RG (|Log2 Fold Change| ≥ 1, *p* < .05). The result showed that 93 proteins were identified, among which 21 proteins were significantly differential abundant between CG and RG as seen in Table 1. It was seen that the abundance of five proteins decreased, which means that these five proteins were extremely sensitive to heat, and measures such as reduction of heat treatment temperature or time taken to reduce the loss of this part of the protein during the actual production process; the abundance of 16 proteins increased, which means that this part of the protein was more stabilized against heat compared to the proteins with reduced abundance.

**TABLE 1:** Significant different proteins by the presence of RG when in comparison with CG

Uniprot ID	Protein name	Log2 Fold change	*p*‐Value	Razor and unique peptides count
O46375	Transthyretin	6.29 ^(+)^	.008	3
G8JKW7	Serpin A3‐7	5.36	.012	3
P34955	Alpha‐1‐antiproteinase	5.15	.007	5
F1MC39	Stromal antigen 1	4.99	.012	1
Q9TTE1	Serpin A3‐1	4.42	.007	3
Q32PA1	CD59 molecule	4.36	.015	1
Q2KJF1	Alpha‐1B‐glycoprotein	3.29	.016	3
Q0IIA2	Odorant‐binding protein‐like	3.07	.037	3
F1MXX6	Lactadherin	2.53	.001	23
E1BGX8	HHIP like 2	1.74	.012	7
Q4GZT4	ATP‐binding cassette subfamily G member 2	1.68	.030	7
P11151	Lipoprotein lipase	1.62	.012	12
A0JNP2	Secretoglobin family 1D member	1.27	.008	2
F1MHI1	Perilipin	1.25	.012	13
F1MZ96	Uncharacterized protein	1.20	.017	5
P79345	NPC intracellular cholesterol transporter 2	1.09	.008	7
P02663	Alpha‐S2‐casein	−1.00^(−)^	.008	14
P61823	Ribonuclease pancreatic	−1.17	.023	3
A0A3Q1MV91	Fibrinogen gamma‐B chain	−1.59	.037	5
P02676	Fibrinogen beta chain	−2.13	.037	11
Q2KIT0	Protein HP‐20 homolog	−3.50	.008	3

Note

(+) represents a significant increase in protein abundance after heat treatment; (−) represents a significant decrease in protein abundance after heat treatment.

We found that the abundance of transthyretin (TTR), serpin A3‐7, alpha‐1‐antiproteinase increased greatly, and TTR had the highest growth abundance (Log2 Fold Change value of TTR is 6, representing a 20‐fold increase in the abundance of TTR) after retort processing. TTR is a protein with a molecular weight of 55 kDa, which can be combined with retinol binding protein and vitamin A complex, and its main role is to participate in the transport of thyroxine (TR) (Adams et al., 2018). It has been reported in the literature that the TTR content in premature infants is lower than that of term infants (Benfield, 2004). Since mammalian TR mainly acts on neuronal cells and Schwann cells in the nervous system, it can stimulate neuronal regeneration and neural function recovery as a whole (Nunes et al., 2005). Therefore, the lower TTR in premature infants may affect the normal development of their nervous system. At the same time, retinol binding protein is used as a carrier protein for vitamin A, and the lack of TTR will directly affect the transport of retinol binding protein, leading to the lack of vitamin A. However, TTR remains poorly understood and the way in which it works in preterm infants seems to be unclear and needs to be explored in further experiments.

It has been reported that serpin A3‐7 can be used to identify potential biomarkers for bovines at risk of metabolic diseases (Almughlliq et al., 2019). Therefore, the phenomenon of serpin A3‐7 abundance changes during retort processes can serve as a “signal amplifier” for the identification of potential biomarkers for bovines at risk. Alpha‐1‐antiproteinase (also called alpha‐1‐antitrypsin or alpha‐1‐proteinase inhibitor) is an acute phase secretory glycoprotein mainly synthesized in hepatocytes (Hazari et al., 2017). The low levels of alpha‐1‐antiproteinase can significantly increase the risk of serious lung or liver disease in children (Torres‐Duran et al., 2018). In present study, we discovered that the abundance of alpha‐1‐antiproteinase increased, which may be beneficial to protect the lung tissue of infants because it can defend lung tissues from damage caused by proteolytic enzymes (Torres‐Duran et al., 2018).

### 3.5 GO analysis of significant different proteins

Gene ontology is a standardized system for describing the characteristics of genes and proteins across different organisms (Bible et al., 2017). The GO describes our knowledge of the biological domain with respect to three aspects: biological process (BP), molecular function (MF), and cellular component (CC). In present study, package of ggplot and Hmisc in RStudio software was used for enrichment of significant different proteins.

**FIGURE 4:**
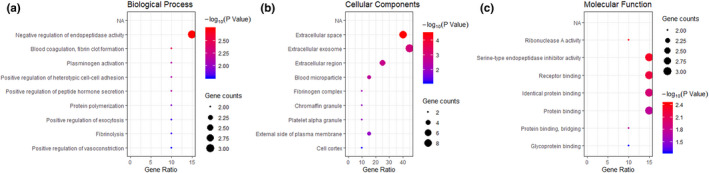
GO analysis for biological process (a), cellular component (b), and molecular function (c) of differential milk serum proteins. The color in the graph represents the high or low *p*‐value, the redder the color the larger the value; the size of the dot represents the gene number, the larger the dot the larger the value

The significant different proteins involved different BPs (Figure 4a) mainly include negative regulation of endopeptidase activity, blood coagulation, fibrin clot formation, and plasminogen activation, where negative regulation of endopeptidase activity is defined as any process that decreases the frequency, rate, or extent of endopeptidase activity, the endohydrolysis of peptide bonds within proteins. Previous study has demonstrated that Emodin could alleviate acute lung injury induced by severe acute pancreatitis via negative regulation of endopeptidase activity and degradation of collagen‐containing extracellular matrix (Xu et al., 2020). In the present study, negative regulation of endopeptidase activity contains three gene counts (gene name, protein name, Uniprot ID: SERPINA3‐7, Serpin A3‐7, G8JKW7; SERPINA1, Alpha‐1‐antiproteinase, P34955; SERPINA3‐1, Serpin A3‐1, Q9TTE1), and protein abundance of all them has increased after retort processing. Unfortunately, we cannot infer what effect this might have on premature infants because BP is not equivalent to a pathway and the GO does not try to represent the dynamics or dependencies that would be required to fully describe a pathway at present (Ashburner et al., 2000; Carbon et al., 2019). The classification of CC is a cellular anatomy, not processes like the other aspects of GO, and the main CCs (Figure 4b) involved extracellular space and extracellular region. In addition, the MFs (Figure 4c) mainly involved ribonuclease A activity, serine‐type endopeptidase inhibitor activity, and receptor binding. Interestingly, serine‐type endopeptidase inhibitor activity contains the same gene counts as negative regulation of endopeptidase activity. The molecular function of serine‐type endopeptidase inhibitor activity mainly stops, prevents, or reduces the activity of serine‐type endopeptidase activity. Since serine proteases and their inhibitors are related to lots of biological processes such as inflammation, coagulation, apoptosis, angiogenesis, and fibrinolysis, further experiments are needed to evaluate the influence of this result on the health of premature infants (Wang et al., 2020).

### 3.6 ELISA validation of the correctness of proteomics results

**FIGURE 5:**
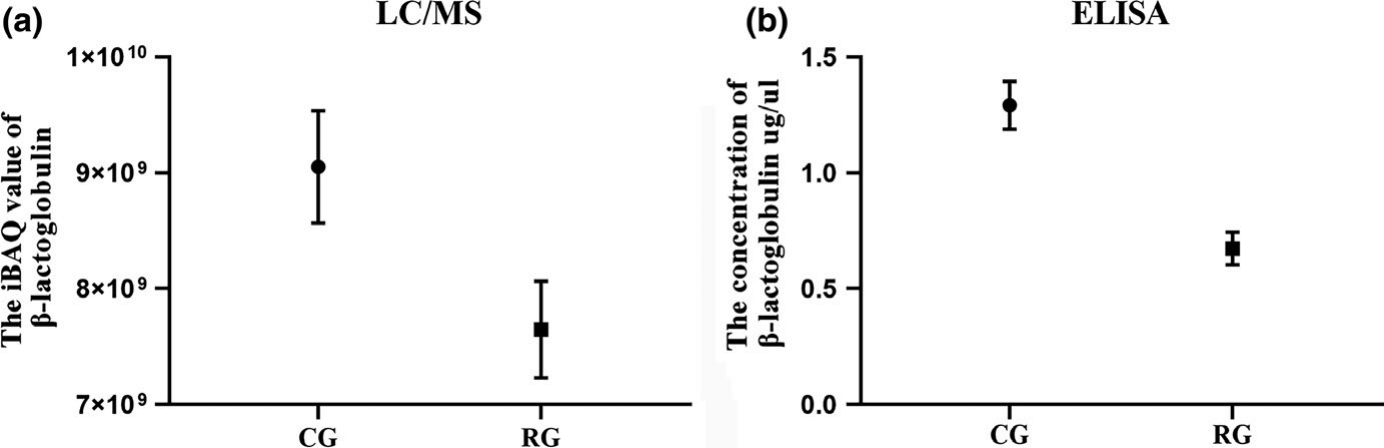
Schematic diagram of β‐lactoglobulin determined by ELISA and LC/MS. The presentation of the values is indicated by mean ± standard deviation (*SD*) of triplicate samples

To verify the correctness of the LC/MS proteomics‐based results, the concentrations of β‐lactoglobulin of CG and RG were determined using ELISA, and the results are shown in Figure 5a. It can be seen that the concentration of β‐lactoglobulin of CG determined by ELISA was higher than that of RG (Figure 5a); the abundance of β‐lactoglobulin of CG determined by proteomics technique was higher than that of RG (Figure 5b). The trend of concentration/abundance of β‐lactoglobulin determined by both methods was in agreement, demonstrating the confidence of the LC/MS‐based proteomics technical tools used in this study.

## 4 CONCLUSION

Retort sterilized milk presents the safest milk available on the market and meets the safety needs of preterm infants. However, the heat treatment of retort sterilization causes loss of milk serum proteins, which plays an important biological role in milk. To obtain information on the changes in bovine milk serum proteins during retort sterilization, we simulated the processing of retort sterilized milk in the laboratory and used LFQ proteomics to investigate the effects of retort sterilization on the raw bovine milk serum proteome. The results of proteomics showed that 96 and 106 proteins were quantified in CG and RG, respectively, and the reason for the greater number of proteins identified in RG than in CG was that heat treatment increased the modifications of bovine milk serum proteins at the molecular level. However, the overall abundance of milk serum proteins (total iBAQ values) identified by RG was less than that of CG, indicating that although heat treatment can increase the digestibility of denatured proteins in raw milk, this improvement was offset by a decrease in the digestibility of proteins caused by protein modifications. The abundance of most of serum proteins in bovine milk was reduced after retort sterilization, but there was also an increase in the abundance of some proteins. The greatest increase in protein abundance was seen in TTR, which may be beneficial to the premature infants’ development of the nervous system and the supplement of vitamin A. However, our state of knowledge of this protein, TTR, is not as sufficient as that of lactotransferrin and lactoperoxidase. Therefore, the conclusions about TTR drawn in this study need to be further explored.

## CONFLICT OF INTEREST

The authors declare that they do not have any conflict of interest.

## ETHICAL STATEMENT

This study does not involve any human or animal testing.

## INFORMED CONSENT

Written informed consent was obtained from all study participants.

## Data Availability

Data are available on request from corresponding author.
